# Prkci acts a pro-proliferation factor in colorectal cancer

**DOI:** 10.1038/s41698-025-01117-y

**Published:** 2025-11-04

**Authors:** Peng Li, Guangshi Liu, Wenbin Zhang, Tao Li

**Affiliations:** 1 Department of Gastrointestinal Surgery, Xinjiang Medical University Affiliated Cancer Hospital, Urumqi, China; 2 Gastrointestinal Surgery Department, People’s Hospital of Xinjiang Uygur Autonomous Region, Xinjiang, Urumqi, China

**Keywords:** Cell biology, Cancer, Gastrointestinal cancer

## Abstract

Colorectal cancer (CRC) is a common malignancy with high mortality, and its treatment remains challenging in advanced stages. Protein kinase C iota (Prkci), a member of the atypical PKC family, is implicated in several cancers, yet its role in CRC is unclear. Here, we report that Prkci is overexpressed in CRC and correlates with poor prognosis. Functional assays showed that Prkci enhances proliferation and metabolic activity, while its knockout suppresses tumor growth both in vitro and in vivo. Mechanistically, Prkci interacts with and phosphorylates c-Myc at serine 21, thereby inhibiting its ubiquitin-mediated degradation and stabilizing the protein. The pro-proliferative effect of Prkci is dependent on c-Myc S21 phosphorylation. In mouse models, deletion of Prkci significantly delayed tumor growth and improved survival. These findings identify Prkci as a key regulator of CRC progression via post-translational stabilization of c-Myc, highlighting it as a potential therapeutic target in colorectal cancer.

## Introduction

Colorectal cancer (CRC) is one of the most common types of cancer worldwide, ranking third in global cancer incidence (9.6% of new cases) and second as a cause of cancer-related mortality (9.3%)^1,2^. Surgical resection, chemotherapy, and radiotherapy are the common treatment methods for localized and advanced colorectal cancer. Recently, targeted therapy and immunotherapy have garnered increasing attention and have achieved promising therapeutic outcomes^3–5^. However, some colorectal cancer patients still experience suboptimal treatment outcomes. Research on colorectal cancer should continue to focus on understanding molecular mechanisms to develop more effective personalized treatment strategies, ultimately improving patient survival rates and quality of life.

Protein kinase C iota (Prkci) is a member of the atypical protein kinase C (aPKC) family, a group of serine/threonine kinases that play roles in cell survival, proliferation, and differentiation^6–9^. Abnormal expression of Prkci has been observed in various cancers, such as ovarian, hepatocellular carcinoma, pancreatic cancer, osteosarcoma, and lung cancer, where it often contributes to tumor progression and poor prognosis. For mechanisms, Prkci positively activates the Akt/mTOR signaling pathway in osteosarcoma; Prkci regulates the activity of the oncogenic transcription factor YAP1 by modulating binding of YAP1 to AMOT130 in ovarian cancer^7,10–12^. However, the role of Prkci in colorectal cancer has not been studied, and this warrants further in-depth investigation, providing potential clues for developing new therapeutic strategies.

Here, we firstly demonstrate that Prkci is significantly upregulated in colorectal cancer, and patients with higher intra-tumoral Prkci expression levels have worse overall survival time. Besides, Prkci positively regulates colorectal cancer cell proliferation. Furthermore, we prove that Prkci phosphorylates c-Myc at serine 21, which obviously blocks the ubiquitin–proteasome pathway degradation of c-Myc. Also, Prkci-mediated colorectal cancer cell proliferation relies on c-Myc S21 phosphorylation. In vivo, knockout of Prkci significantly suppresses colorectal cancer growth and prolongs the overall survival time of mice. Overall, our findings imply that Prkci might be an effective treatment target for colorectal cancer.

## Results

### High Prkci expression in colorectal cancer correlated with poor prognosis

**Fig. 1:**
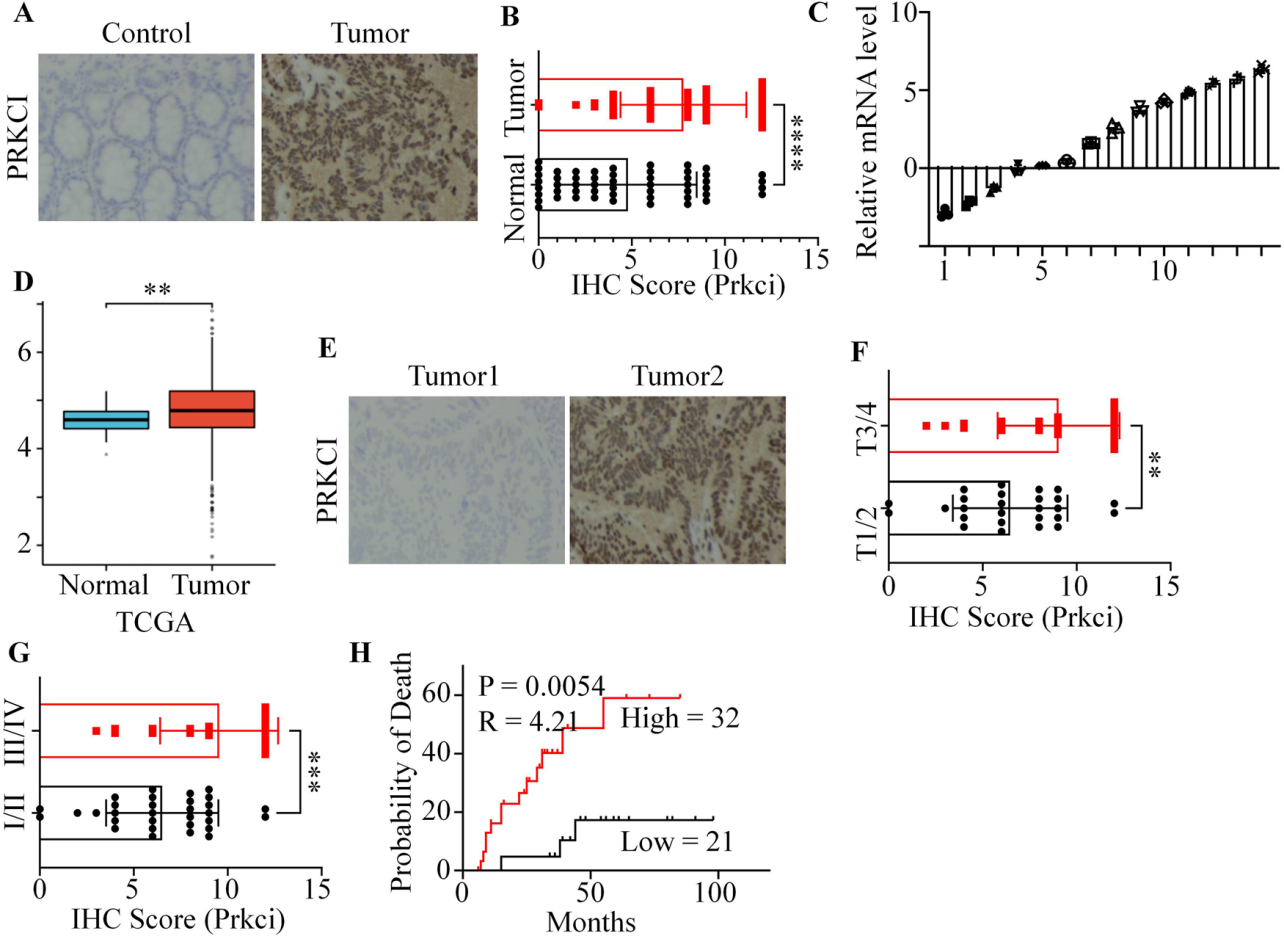
Prkci expression is elevated in colorectal cancer and correlates with tumor progression and patient survival. **A** Immunohistochemical (IHC) staining of Prkci in colorectal cancer (CRC) and adjacent normal tissues. **B** Quantitative analysis of Prkci expression levels in CRC versus normal tissues. **C** Relative Prkci mRNA levels in normal and tumor tissues. **D** Analysis of Prkci expression in CRC tissues using The Cancer Genome Atlas (TCGA) dataset. **E** IHC of Prkci in different tumors. **F** Comparison of Prkci IHC scores by clinical T stage, demonstrating increased Prkci expression in higher stages. **G** Comparison of Prkci IHC scores by clinical stage, demonstrating increased Prkci expression in higher stages. **H** Overall survival analysis of patients grouped by Prkci expression levels, indicating that high Prkci expression correlates with poorer survival. Each IB assay was performed in triplicate, yielding consistent results. Statistical analysis was conducted using Student’s *t* test.

To reveal the biological function of Prkci in colorectal cancer, we collected 53 pairs of colorectal cancer tissues and adjacent normal tissues. Immunohistochemical (IHC) staining revealed that Prkci expression was significantly higher in colorectal cancer tissues compared to adjacent normal tissues (Fig. 1A, B). Quantitative analysis of clinical samples (*n* = 14) indicated that Prkci expression was markedly increased in tumor tissues (Fig. 1C). Analysis using the TCGA dataset confirmed this observation, showing that Prkci was overexpressed in colorectal cancer tissues than that in normal colorectal tissues (Fig. 1D). Of note, Prkci was also overexpressed in other kinds of cancer (Fig. S1A). Subsequently, patients were grouped by clinical T stage or pathological stage. IHC analysis showed that high Prkci expression was associated with advanced tumor stages (Fig. 1E–G). Next, we divided patients into two groups according to the IHC score. IHC scores of 7 and above were classified as the high-expression group, while others were classified as the low-expression group. Survival analysis showed that patients with high IHC scores were associated with worse overall survival (Fig. 1H). Survival analysis presented similar results in other kinds of cancer using TCGA databases (Fig. S1B).

### Prkci positively promoted colorectal cancer cell proliferation and metabolism

**Fig. 2:**
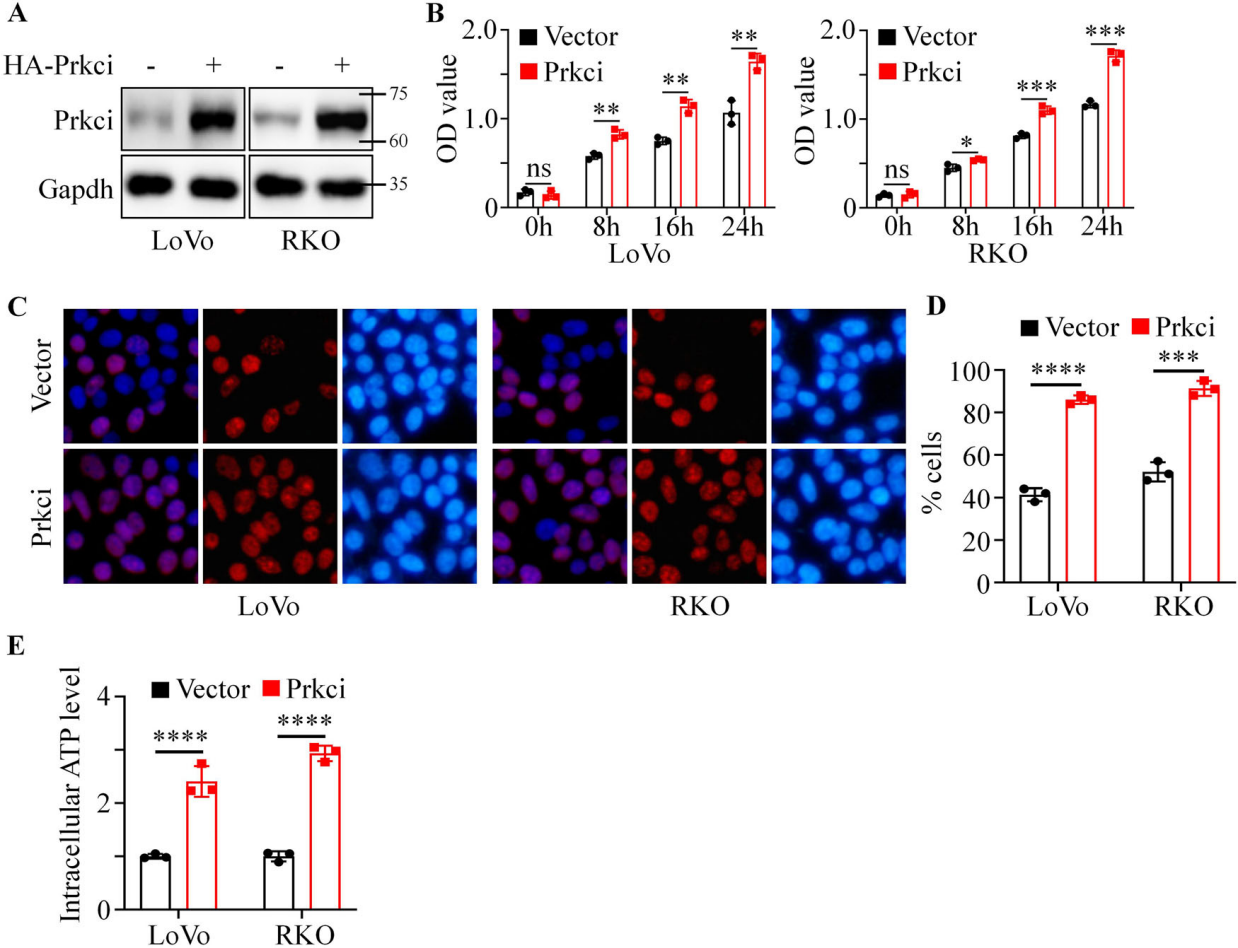
Prkci overexpression promotes colorectal cancer cell proliferation and enhances metabolic activity. **A** Western blot confirmation of Prkci overexpression in LoVo and RKO CRC cell lines. **B** CCK8 assay showing increased proliferation in Prkci-overexpressing cells. **C**, **D** BrdU assay indicating enhanced DNA synthesis in Prkci-overexpressing cells. **E** Intracellular ATP levels in vector control and Prkci-overexpressing cells, demonstrating higher metabolic activity in the latter. Each IB assay was performed in triplicate, yielding consistent results. Statistical analysis was conducted using Student’s *t* test.

To further investigate the functional impact of Prkci in colorectal cancer, we established stable Prkci-overexpressing LoVo and RKO cell lines using lentivirus. Western blotting confirmed the successful construction of the cell lines (Fig. 2A). CCK8 assays demonstrated that Prkci overexpression significantly promoted cell proliferation compared to control cells (Fig. 2B). Next, we carried out Brdu assays to mark cells in the S phase. Results showed that a higher proportion of cells is in the S phase in Prkci overexpression cells, which implied that Prkci overexpression cells had a higher ability to replicate DNA (Fig. 2C, D). Then, we assessed the cellular metabolism ability of vector and Prkci cells. Overexpression of Prkci led to a significant increase in intracellular ATP levels, indicating enhanced metabolic activity (Fig. 2E).

**Fig. 3:**
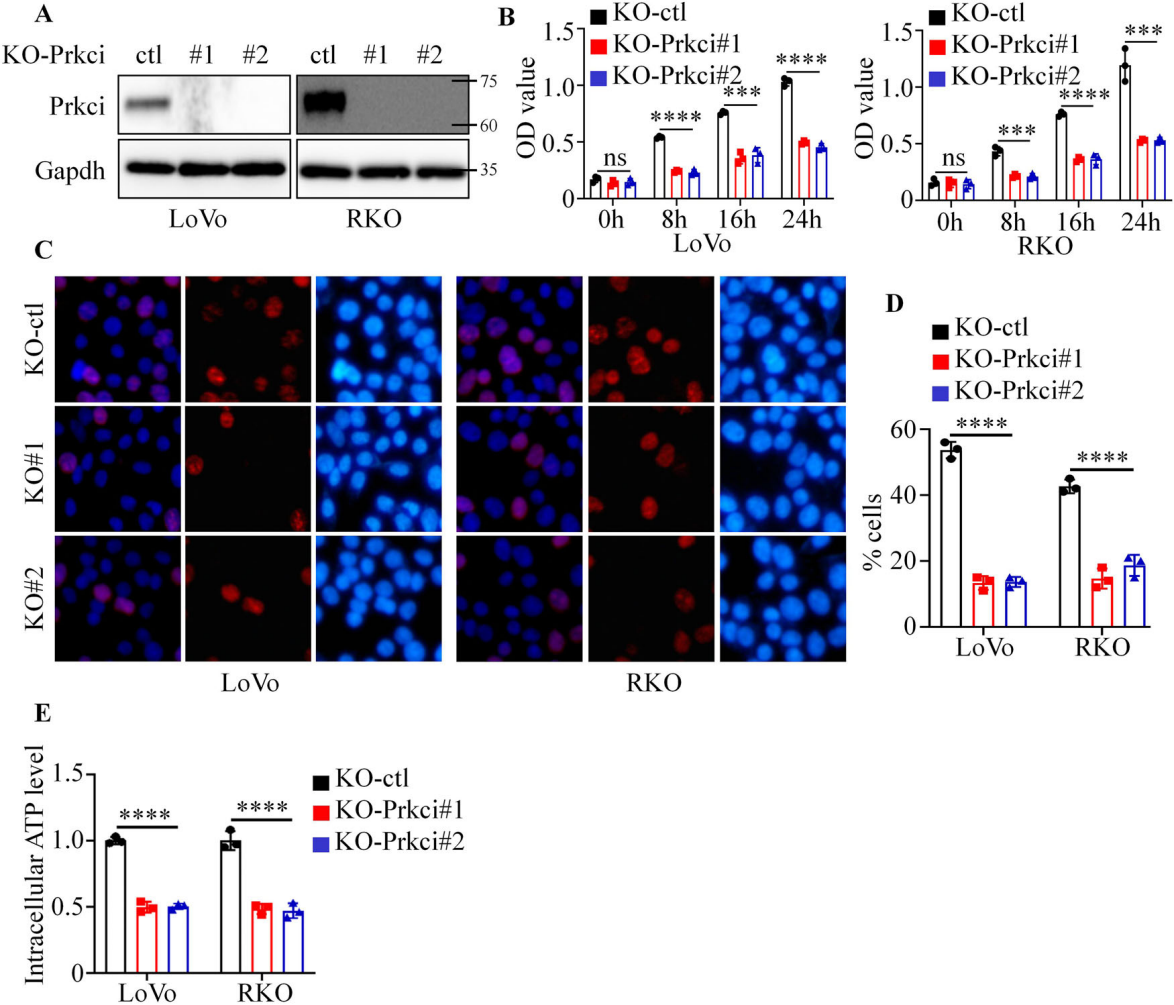
Prkci knockout reduces proliferation and metabolic activity in colorectal cancer cells. **A** Western blot showing Prkci knockout in LoVo and RKO cells using CRISPR-Cas9. **B** CCK8 assay results showing reduced cell proliferation following Prkci knockout. **C**, **D** BrdU assay displaying decreased S-phase entry in Prkci knockout cells. **E** Intracellular ATP measurements, indicating a decline in metabolic activity upon Prkci knockout. Each IB assay was performed in triplicate, yielding consistent results. Statistical analysis was conducted using one-way ANOVA test (KO-ctl vs. KO-Prkci#1, KO-ctl vs. KO-Prkci#2). The significance bar indicates the largest *P* value among the comparisons.

Next, we knocked out Prkci using the CRISPR-Cas9 system, and western blotting confirmed the successful construction of the cell lines (Fig. 3A). Consistently, knocking out Prkci markedly reduced cell growth, as confirmed by CCK8 and BrdU incorporation assays (Fig. 3B–D). Cells with Prkci knockout exhibited a reduction in ATP levels, supporting the role of Prkci in cellular energy metabolism (Fig. 3E).

### Prkci stabilized c-Myc and promoted its expression

**Fig. 4:**
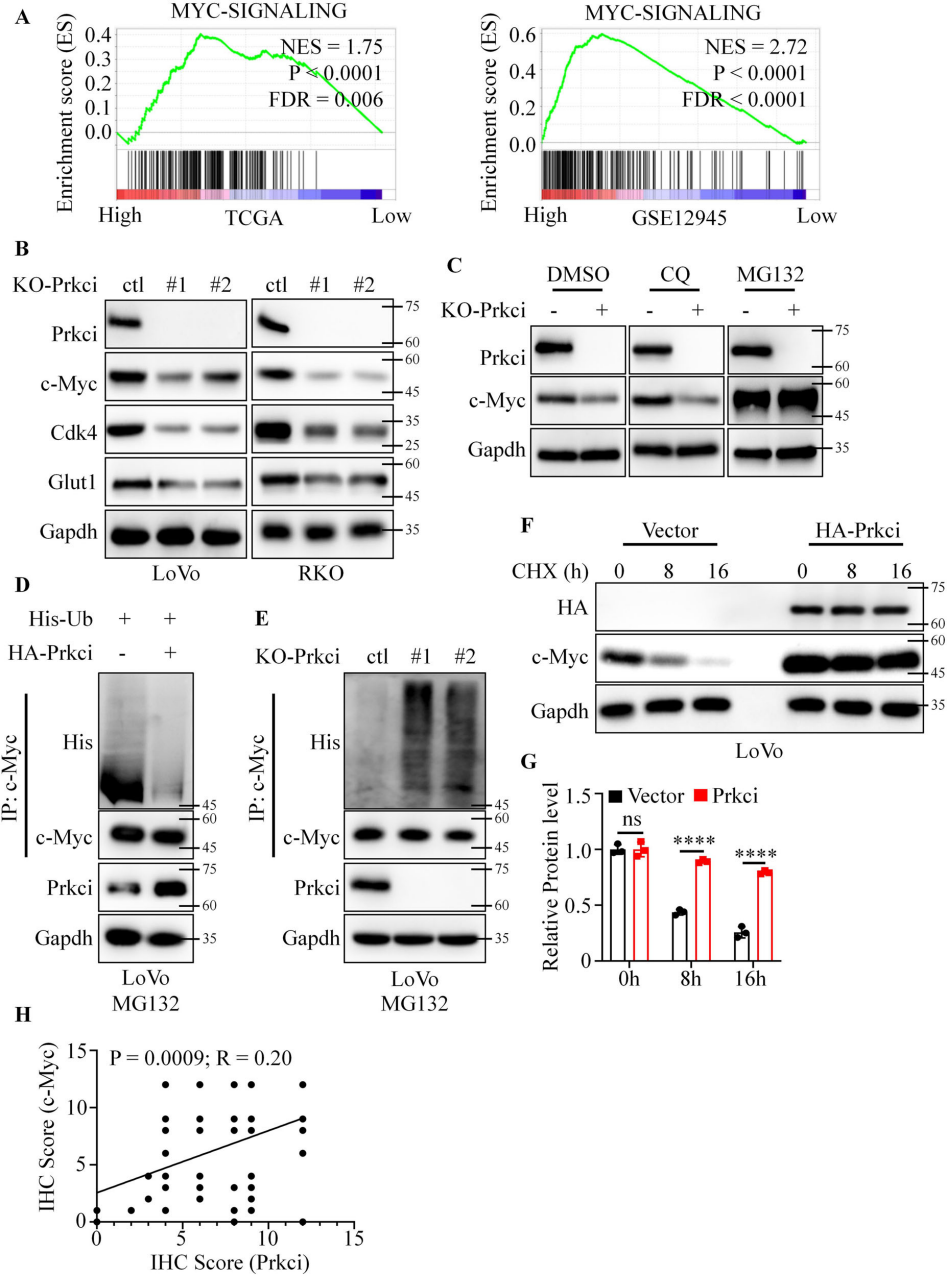
Prkci stabilizes and upregulates c-Myc in colorectal cancer cells. **A** Gene Set Enrichment Analysis (GSEA) highlighting activation of c-Myc signaling pathways associated with high Prkci expression. **B** Western blot analysis showing elevated c-Myc, Cdk4, and Glut1 protein levels in Prkci knockout cells. **C** Effect of CQ and MG132 on c-Myc levels in Prkci knockout cells. **D**, **E** Polyubiquitination levels of c-Myc in control and Prkci-altered cells. **F**, **G** Cycloheximide chase assay displaying slower c-Myc degradation rate with Prkci overexpression. **H** Statistical correlation analysis of Prkci and c-Myc expression levels in CRC tissues. Each IB assay was performed in triplicate, yielding consistent results. Statistical analysis was conducted using Student’s *t* test.

Gene Set Enrichment Analysis (GSEA) of the TCGA dataset indicated that high Prkci expression was associated with the activation of c-Myc signaling pathways (Fig. 4A). This was validated across multiple independent datasets (Fig. 4A and Fig. S2A). Western blot analysis showed that Prkci overexpression significantly elevated c-Myc protein levels in both LoVo and RKO cells (Fig. S2B). Moreover, downstream targets of c-Myc, such as Glut1 and Cdk4, were also upregulated in Prkci-overexpressing cells (Fig. S2B). Consistently, Prkci knockout cells displayed decreased c-Myc and its downstream proteins levels (Fig. 4B and Fig. S2C). Interestingly, Prkci had no effect on the mRNA level of c-Myc, implying that Prkci influenced the rate of protein degradation (Fig. S3A). Previous reports had proved that autophagy–lysosome and ubiquitin–proteasome were the two main ways of protein degradation^13,14^. Therefore, we applied chloroquine (CQ), a lysosome inhibitor, and MG132, a proteasome inhibitor, in KO-ctl and KO-Prkci cells. Western blotting confirmed that MG132 significantly reversed that knock-out of Prkci decreased the cellular c-Myc level (Fig. 4C). Then, we detected the poly-ubiquitin level of c-Myc in vector, Prkci, KO-ctl, and KO-Prkci cells. We first treated cells with MG132 to inhibit endogenous c-Myc degradation. Western blotting confirmed that Prkci positively regulated the poly-ubiquitin level of c-Myc (Fig. 4D, E). Cycloheximide (CHX) was used to suppress endogenous c-Myc synthesis, and western blotting confirmed that Prkci overexpression led to a significant decrease in the degradation rate of c-Myc (Fig. 4F, G). Also, we analyzed the correlation between Prkci and c-Myc expression level using IHC assays, which demonstrated that there was a positive relationship between Prkci and c-Myc expression level (Fig. 4H and Fig. S3B).

### Prkci modulated c-Myc phosphorylation to enhance stability

**Fig. 5:**
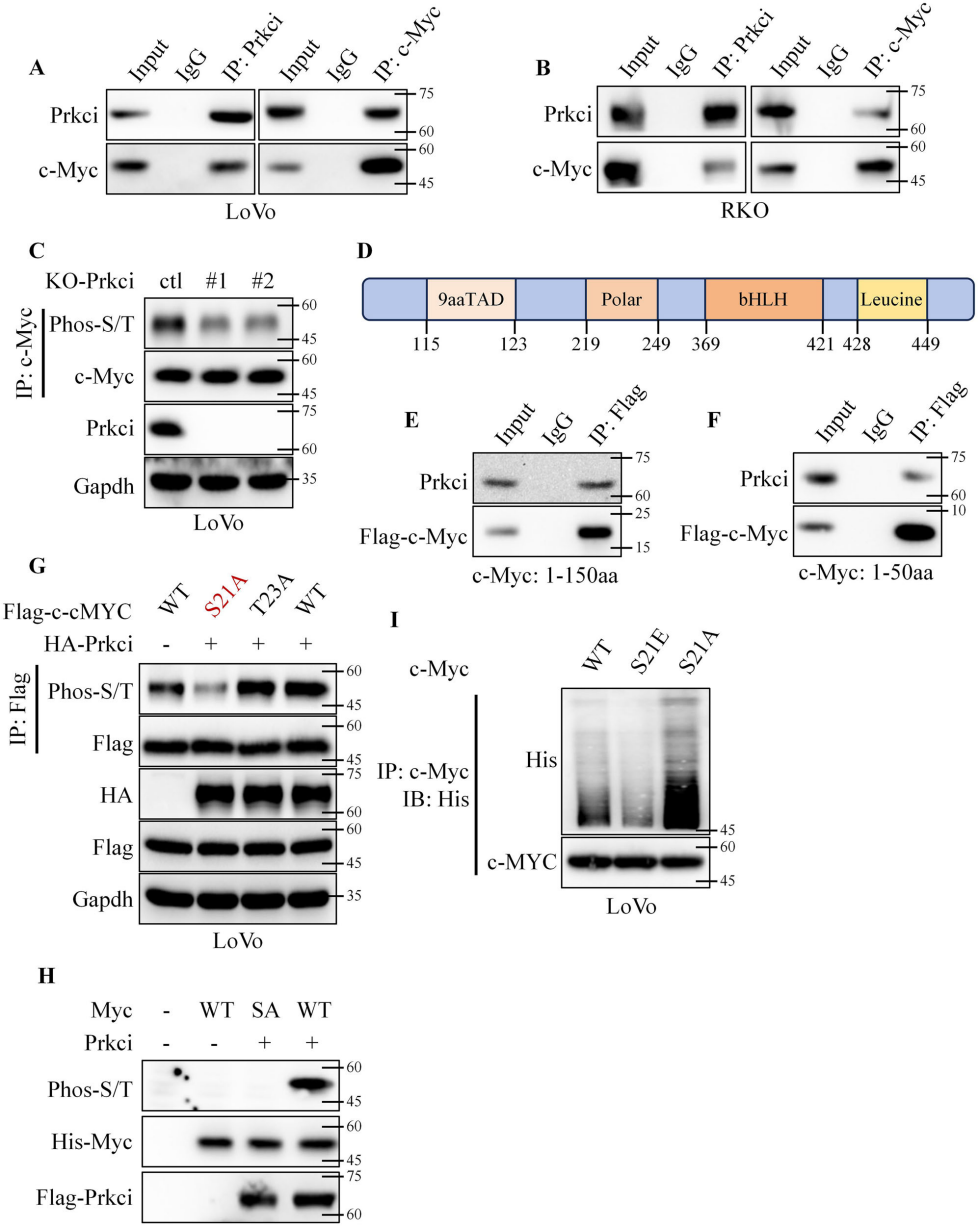
Prkci interacted with and induced phosphorylation of c-Myc in colorectal cancer cells. **A**, **B** Co-immunoprecipitation (Co-IP) of Prkci with c-Myc, confirming their interaction. **C** Western blot analysis of the phosphorylation level of c-Myc in Prkci knockout cells. **D** Schematic of c-Myc domains. **E** Co-IP of Prkci with c-Myc 1–150 aa segments, identifying the main interaction region. **F** Co-IP of Prkci with c-Myc 1–50 aa segments, identifying the main interaction region. **G** Western blot analysis of Prkci-mediated phosphorylation of c-Myc at serine 21. **H** In vitro kinase assay using Flag-Prkci protein, His-tagged c-Myc WT and S21A mutant proteins, followed by western blot. **I** Western blot showing effects of phosphorylation-inactive (S21A) and phosphomimetic (S21E) mutations of c-Myc on the polyubiquitination levels of c-Myc. Each IB assay was performed in triplicate, yielding consistent results.

Subsequently, immunoprecipitation (IP) assays revealed that Prkci interacted with c-Myc (Fig. 5A, B). Considering that Prkci was a serine/ threonine-protein kinase, we assumed that Prkci might phosphorylated c-Myc^6^. Then, we used the pan Phospho-Serine/Threonine antibody to detect the phosphorylation level of c-Myc in vector, Prkci, KO-ctl, and KO-Prkci cells. Western blotting confirmed that Prkci positively increased the phosphorylation of c-Myc at specific serine/threonine residues (Fig. 5C and Fig. S4A). Subsequently, we further analyzed which amino acid of c-Myc was phosphorylated by Prkci. We firstly constructed segmented plasmids according to the domains of c-Myc (Fig. 5D). Co-immunoprecipitation experiments identified the 1–50 amino acid region of c-Myc as the primary interaction site for Prkci (Fig. 5E, F and Fig. S4B, C). After browsing the PhosphoSitePlus database, we found that there were two potential serine/threonine phosphorylation sites (S21 and T23). Next, the two phosphorylation sites were mutated to alanine, respectively, a kind of phosphorylation-inactive mutation. Western blotting and IP assays confirmed that S21A mutant abrogated Prkci-mediated c-Myc phosphorylation, which proved that Prkci phosphorylated c-Myc at S21 (Fig. 5G). In addition, we isolated Flag-tagged Prkci protein from HEK293T cells and purified His-tagged c-Myc WT and S21A mutant proteins using a prokaryotic expression system. In vitro kinase assays demonstrated that Prkci could phosphorylate His–c-Myc WT, but not the S21A mutant, indicating that Prkci specifically phosphorylates c-Myc at serine 21 (Fig. 5H). Subsequently, we knocked out endogenous c-MYC expression in LoVo cells and reintroduced either wild-type c-MYC (WT), S21A, or S21E (mutated serine to glutamic acid (E), a kind of phosphomimetic mutation) mutant to generate stable cell lines (Fig. S4D, E). Western blotting proved that c-Myc S21 phosphorylation blocked its polyubiquitin (Fig. 5I).

### Prkci-mediated colorectal cancer cell proliferation relied on c-Myc phosphorylation

**Fig. 6:**
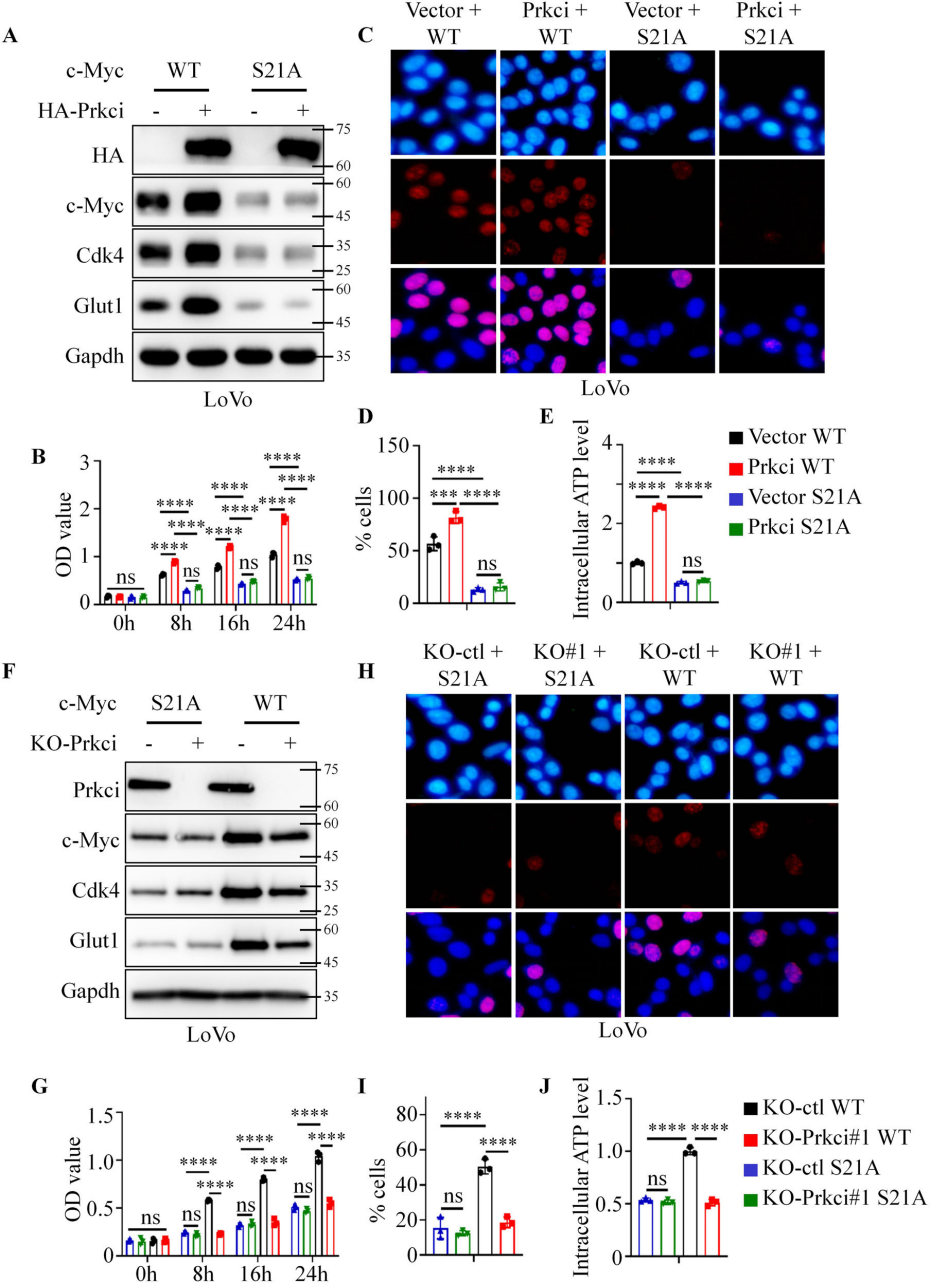
Prkci-mediated colorectal cancer cell proliferation depends on c-Myc phosphorylation. **A**, **F** Western blot showing expression levels of c-Myc and downstream targets (Cdk4, Glut1) in various cell conditions, including wild-type and phosphorylation-inactive c-Myc. **B**, **G** CCK8 assay demonstrating the effect of Prkci and c-Myc S21A mutations on cell proliferation. **C**, **D**, **H**, **I** BrdU assay showing S-phase entry under different Prkci and c-Myc conditions. **E**, **J** Intracellular ATP measurements illustrating metabolic activity under different Prkci and c-Myc conditions. Each IB assay was performed in triplicate, yielding consistent results. Statistical analysis was conducted using a one-way ANOVA test.

To further clarify the relationship between Prkci and c-Myc in colorectal cancer cell proliferation, we used the cell line from Fig. 5I to construct four kinds of LoVo cells stably expressing vector + c-Myc WT, Prkci + c-Myc WT, vector + c-Myc S21A, Prkci + c-Myc S21A, respectively. c-Myc S21A cells had lower c-Myc, Cdk4 and Glut1 expression than c-Myc WT cells (Fig. 6A). Meanwhile, Prkci overexpression upregulated c-Myc, Cdk4, and Glut1 expression in c-Myc WT cells, but not in c-Myc S21A cells (Fig. 6A). Also, c-Myc S21A cells had lower proliferation and cellular energy metabolism ability than c-Myc WT cells, and Prkci overexpression enhanced the proliferation and cellular energy metabolism ability in c-Myc WT cells, but not in c-Myc S21A cells (Fig. 6C–E). Next, we used the cell line from Fig. 5I to further construct KO-ctl + c-Myc WT, KO-Prkci#1 + c-Myc WT, KO-ctl + c-Myc S21A, KO-Prkci#1 + c-Myc S21A, respectively. Consistently, knock-out of Prkci could inhibit c-Myc, Cdk4 and Glut1 expression, cell proliferation, and cellular energy metabolism in c-Myc WT cells, but not in c-Myc S21A cells (Fig. 6H–J).

### Targeting Prkci inhibited tumor growth in vivo

**Fig. 7:**
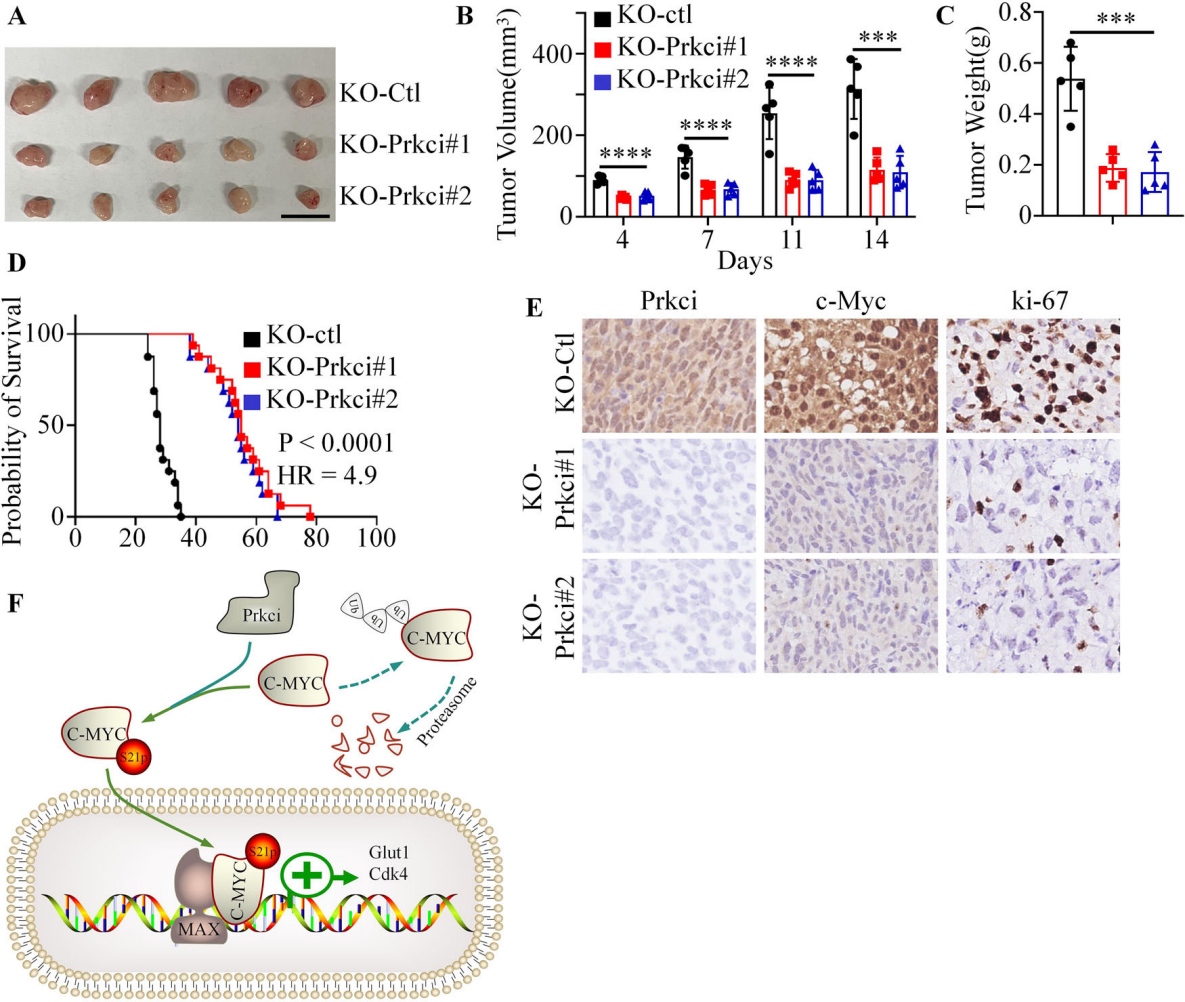
Prkci knockout inhibits tumor growth and improves survival in a colorectal cancer mouse model. **A** Tumor image is shown. **B** Tumor volume in control and Prkci knockout CRC xenografts. **C** Tumor weights, showing significantly smaller tumors in the Prkci knockout group. **D** Kaplan–Meier survival analysis of mice with control and Prkci knockout tumors. **E** Representative IHC staining of c-Myc and Ki-67 in tumor tissues from control and Prkci knockout mice, showing reduced proliferation markers in Prkci-deficient tumors. Each IB assay was performed in triplicate, yielding consistent results. Statistical analysis (**B**, **C**) was conducted using one-way ANOVA test (KO-ctl vs. KO-Prkci#1, KO-ctl vs. KO-Prkci#2). The significance bar indicates the largest *P* value among the comparisons. **F** Prkci phosphorylated c-Myc at serine 21, and promoted tumor growth.

To assess the impact of Prkci on tumorigenesis, LoVo cells with Prkci knockout were injected into the back of nude mice. Tumor volume was measured every three days. Tumors derived from Prkci KO cells displayed significantly slower growth, smaller volumes, and lighter weight compared to control tumors (Fig. 7A–C). Kaplan–Meier survival analysis revealed that mice bearing Prkci KO tumors had significantly improved survival (Fig. 7D). IHC analysis of excised tumors showed a marked decrease in c-Myc and Ki-67 levels in the Prkci KO group, suggesting reduced proliferation in the absence of Prkci (Fig. 7E).

## Discussion

In this study, we identified Prkci as a key regulator of colorectal cancer (CRC) proliferation via its interaction with c-Myc, a well-known oncogenic transcription factor. Our results demonstrate that Prkci promotes CRC growth by stabilizing c-Myc through phosphorylation at serine 21, thereby enhancing c-Myc’s resistance to ubiquitin–proteasome-mediated degradation. This mechanism elucidates a novel role for Prkci in CRC and positions it as a potential therapeutic target. Our findings align with previous studies suggesting the broader oncogenic role of Prkci across various cancer types, including osteosarcoma, pancreatic cancer, and cervical cancer. Although previous studies have shown that Prkci is critical for embryonic development, particularly for the establishment of the maternal–fetal exchange interface during placental formation, it is noteworthy that viable Prkci knockout mice have been successfully generated and are commercially available (e.g., C57BL/6JCya-Prkciem1/Cya, Cyagen)^15^. This indicates that under specific genetic contexts or with certain knockout strategies, PRKCI loss may be at least partially tolerated after birth. Moreover, PRKCI is frequently overexpressed in multiple human cancers, including colorectal, lung, ovarian, and pancreatic cancers, whereas its expression is relatively low in most normal adult tissues^16^. This cancer-specific expression pattern suggests the existence of a therapeutic window, allowing for selective inhibition of PRKCI in tumor cells with potentially minimal toxicity to normal tissues. Although aurothiomalate (ATM) has been explored as a Prkci inhibitor in several cancers, it mainly disrupts PB1-domain-mediated interactions rather than inhibiting the kinase domain^17^. Since our study reveals that Prkci exerts its oncogenic effects in CRC through direct phosphorylation of c-Myc, the development of kinase-specific inhibitors would be a more rational therapeutic strategy. Currently, such inhibitors are not available, but our findings highlight the potential value of pursuing this direction in future drug discovery. Together, these findings support the feasibility of targeting PRKCI in cancer treatment, while also underscoring the need for further investigation into its physiological roles and on-target safety in preclinical settings.

The stabilization of c-Myc through Prkci-mediated phosphorylation provides insight into how Prkci influences cellular processes essential for tumorigenesis. c-Myc’s role in promoting cellular proliferation, metabolism, and survival is well-documented, particularly through its regulation of genes involved in glycolysis, cell cycle progression, and apoptosis^18,19^. The interplay between Prkci and c-Myc thus extends beyond CRC and may underlie tumor progression in other cancers as well. For instance, Prkci has been shown to contribute to radio-resistance in cervical cancer by activating the Hedgehog/GLI1 pathway, enhancing cancer cell survival under treatment^7^. In CRC, Prkci appears to use a similar mechanism of augmenting the stability and activity of oncogenic factors like c-Myc, which could explain its association with poor prognosis in CRC patients, as observed in our study.

Furthermore, previous research has highlighted the role of Prkci in promoting tumor growth and metastasis in pancreatic cancer via its interaction with the kinase RIPK2, leading to increased phosphorylation of NF-κB, JNK, and ERK pathways^11^. This finding, together with our demonstration that Prkci activates the c-Myc pathway, suggests that Prkci is involved in multiple oncogenic signaling cascades that contribute to tumor aggression and resilience. Notably, these pathways, including Akt/mTOR in osteosarcoma, are interconnected, and Prkci’s role as a serine/threonine kinase may facilitate crosstalk between them^10^. The identification of Prkci as a central node in these pathways underscores its potential as a target for multifaceted cancer therapies.

Our study also reinforces the challenges of targeting c-Myc, often regarded as an “undruggable” target due to its structure and role as a universal amplifier of transcription^20,21^. By focusing on Prkci, which regulates c-Myc post-translationally, we may overcome some limitations of directly targeting c-Myc itself. Strategies that inhibit Prkci could indirectly destabilize c-Myc, reducing its oncogenic activity in CRC and potentially other cancers where Prkci plays a regulatory role. It is worth noting that c-Myc is a well-established transcriptional target of the APC-Wnt signaling pathway, which represents a key oncogenic driver in colorectal cancer progression^22–24^. However, our study suggests that Prkci regulates c-Myc at the post-translational level through serine 21 phosphorylation, thus stabilizing the protein and enhancing its oncogenic effects independently of upstream transcriptional activation. This raises the possibility that Prkci functions as a parallel or synergistic amplifier of c-Myc activity in CRC. Future studies are warranted to determine whether Prkci is itself regulated by upstream CRC pathways or whether it represents a bypass route to sustain c-Myc activity even in the absence of canonical pathway activation.

Although the mutation status of APC, KRAS, and BRAF was not available for the patient specimens used in this study, our in vitro models - LoVo (harboring APC and KRAS mutations), RKO (harboring a BRAF mutation), and SW48 (wild-type for APC, KRAS, and BRAF) - demonstrated that Prkci stabilizes c-Myc regardless of these upstream oncogenic alterations. Given that mutations in APC, KRAS, and BRAF are among the most frequently occurring genetic alterations in colorectal cancer, our findings suggest that the Prkci–c-Myc axis may represent a generalizable and mutation-independent oncogenic mechanism in CRC.

In conclusion, our findings suggest that Prkci promotes CRC progression by stabilizing c-Myc through phosphorylation, thereby enhancing its oncogenic potential. Given Prkci’s role across diverse cancer types, further research into Prkci inhibitors could yield valuable therapeutic agents capable of disrupting key pathways in cancer progression.

## Methods

### Antibodies and reagents

Antibodies for Prkci (66493, Proteintech), Gapdh (ab181602, Abcam), Cdk4 (ab108357, Abcam), Glut1 (66290, Proteintech), HA-tag (66006, Proteintech), His-tag (66005, Proteintech), Flag-tag (66008, Proteintech), and pan Phospho-Serine/Threonine (AP1475, Abclonal) were purchased from commercial companies. Chloroquine (HY-17589A) and MG-132 (HY-13259) were bought from the MedChemExpress company.

### Cell lines

RKO and LoVo cell lines are purchased from the American Type Culture Collection (ATCC). The base medium for RKO is Eagle’s Minimum Essential Medium, while the base medium for LoVo is F-12K medium. All media are added with 10% fetal bovine medium. The cells are cultured in 37 °C, 95% air, 5% CO_2_.

### Cell viability analysis

Cell Counting Kit-8 (RM02823) is bought from ABclonal. The cell viability analysis is applied according to the instructions.

### Intracellular ATP analysis

General Adenosine Triphosphate (ATP) ELISA Kits (RK04252) are purchased from ABclonal. The intracellular ATP level is detected according to the instructions.

### Real-time PCR assays

Total RNA was extracted from samples using TRIzol reagent, and concentration and purity were verified (A260/A280 = 1.8–2.0). One microgram of RNA was reverse-transcribed into cDNA using the PrimeScript RT Reagent Kit (RK21400, ABclonal). qPCR was performed in a 20 µL reaction volume containing 10 µL of SYBR Green Master Mix (RK21203, ABclonal). Reactions were run on an ABI 7500 Real-Time PCR System. Relative quantification was performed using the ΔΔCt method, with GAPDH as the reference gene. The primers are listed as follows: Prkci F: 5;-AGGTCCGGGTGAAAGCCTA-3, Prkci R: 5-TGAAGAGCTGTTCGTTGTCAAA-; Myc F: 5-GTCAAGAGGCGAACACACAAC-3, Myc R: 5-TTGGACGGACAGGATGTATGC-3.

### Western blotting and immunoprecipitation

Proteins are extracted from samples and quantified using the BCA assay. Equal amounts of protein are loaded onto SDS-PAGE gels for electrophoresis, then transferred onto PVDF membranes. Membranes are blocked with 5% non-fat milk in TBST for 1 h at room temperature, followed by overnight incubation with primary antibodies at 4 °C. After washing, membranes are incubated with HRP-conjugated secondary antibodies for 1 h at room temperature. Protein bands are visualized using enhanced chemiluminescence (ECL) and captured on an imaging system. Relative band intensities are quantified using GAPDH as the loading control. For immunoprecipitation, the lysates are incubated with a target-specific primary antibody for 2–4 h at 4 °C with gentle rotation, followed by the addition of Protein A/G agarose or magnetic beads, incubating for another 1–2 h to capture the antibody-protein complexes. Beads are then washed multiple times with lysis buffer to remove nonspecific proteins. Bound proteins are eluted by adding SDS sample buffer, boiled, and analyzed by SDS-PAGE and Western blotting to detect the immunoprecipitated proteins.

### Cell lines construction

CRISPR/Cas9-mediated gene knockout was performed using the plasmid pSpCas9(BB)-2A-Puro (PX459) V2.0. Guide RNAs (sgRNAs) targeting the human Prkci and c-MYC genes were designed as follows: sgPRKCI#1: 5’-TTAAATTATCTTCATGA-3; sgPRKCI#2: 5-TAAATTATCTTCATGAG-3; sgPRKCI#3: 5-CACTGACTACGGCATGT-3; sgMYC#1: 5-TTGAGGGGCATCGTCGC-3; sgMYC #2: 5- ACGTTGAGGGGCATCGT-3; sgMYC #3: 5-AACGTTGAGGGGCATCG-3. sgRNAs were cloned into the PX459 V2.0 vector following the standard protocol. Cells were transfected with the recombinant PX459-sgRNA plasmids. At 48 h post-transfection, cells were subjected to puromycin (2 μg/mL) selection for 5–7 days to enrich for successfully transfected cells. Single colonies were isolated, expanded, and screened for knockout efficiency by PCR genotyping and Western blot analysis. Knockout clones with complete loss of protein expression were selected for subsequent experiments.

To generate stable cell lines with high expression of target genes, a lentiviral packaging system was used. Briefly, the pLVX expression vector containing the target gene was co-transfected with PPAX2 and MD2.G helper plasmids into HEK293T cells. After 48–72 h, the viral supernatants were collected, filtered (0.45 μm), and used to infect target cells. Infected cells were selected with puromycin (2 μg/mL) for 5–7 days to establish stable cell lines with high expression of the target gene, which was further confirmed by qRT-PCR and western blot.

### Immunohistochemistry (IHC)

This research involving human subjects was approved by the Ethical Committee of People’s Hospital of Xinjiang Uygur Autonomous Region, with written informed consent obtained from all participants, and all procedures were conducted in accordance with the ethical principles of the Declaration of Helsinki. Tissue sections are deparaffinized in xylene and rehydrated through graded alcohols. Antigen retrieval is performed by heating sections in citrate buffer (pH 6.0). Sections are then blocked with 5% BSA to prevent nonspecific binding and incubated with primary antibodies overnight at 4 °C. After washing, sections are incubated with an HRP-conjugated secondary antibody for 1 h at room temperature. Signals are developed using DAB substrate for chromogenic detection or visualized directly for fluorescence, and sections are counterstained with hematoxylin before mounting for microscopic analysis. The IHC scoring is assessed as follows: The intensity score ranges from 0 to 3, where 0 indicates no staining, 1 is weak staining, 2 is moderate staining, and 3 is strong staining. The proportion of positive cells is scored based on the percentage of cells stained, ranging from 0 (0–10%) to 4 (76–100%). A total score is often calculated by multiplying or adding the intensity and percentage scores, with higher scores indicating stronger expression.

### Brdu assays

Cells were seeded in culture plates and incubated with BrdU (10 µM; A1482, ABclonal) for 2–4 h, allowing sufficient time for incorporation during the S-phase of the cell cycle. Following BrdU treatment, cells were fixed with 4% paraformaldehyde for 20 min at room temperature. Cells were then incubated with a mouse anti-BrdU primary antibody (1:100) overnight at 4 °C. The next day, cells were incubated with a fluorescently labeled secondary antibody (Alexa Fluor 594; AS054, ABclonal) for 1 h at room temperature. Nuclei were counterstained with DAPI. BrdU-positive nuclei were visualized using a fluorescence microscope.

### In vitro kinase assay

Recombinant Flag-Prkci protein was immunopurified from transfected HEK293T cells using anti-Flag magnetic beads. His-tagged c-Myc WT and S21A mutant proteins were expressed in *E. coli* BL21 (DE3) and purified using Ni-NTA agarose resin under native conditions. For the kinase reaction, 1 µg of His–c-Myc WT or S21A protein was incubated with 0.5 µg of Flag-Prkci in kinase buffer (25 mM Tris-HCl pH 7.5, 10 mM MgCl₂, 1 mM DTT, 100 µM ATP) in a total volume of 30 µL at 30 °C for 30 min. The reaction was terminated by adding SDS loading buffer and boiling for 5 min.

Samples were resolved by SDS-PAGE and analyzed by immunoblotting.

### Xenograft assay

This study was reported in accordance with the ARRIVE guidelines. A completed ARRIVE Essential 10 checklist is provided as Supplementary Information. Animal studies were conducted in compliance with protocols approved by the Institutional Animal Care and Use Committee at People’s Hospital of Xinjiang Uygur Autonomous Region. Mice were randomly assigned to different experimental groups. SW48 cells (5 × 10^6^ cells) were resuspended in a 100 µL mixture of PBS and Matrigel (1:1) and injected subcutaneously into the flank of 6–8-week-old BALB/c nude mice. Tumor growth was monitored by measuring the tumor size with calipers every 3 days. Tumor volume was calculated using the formula: Volume = (length × width^2^)/2. At the end of the experiment, mice were euthanized using CO₂ inhalation in a gradual-fill chamber, and tumors were excised for further analysis. All animal procedures were conducted according to institutional guidelines and approved by the animal ethics committee of People’s Hospital of Xinjiang Uygur Autonomous Region.

### GSEA analysis

GSEA was performed using the GSEA software (v4.3.2) with standard settings. TCGA-COAD and GEO datasets (GSE12945, GSE17536, GSE29623, GSE31595, GSE75535, GSE87211, GSE106535, GSE133057) were stratified into PRKCI-high and PRKCI-low groups based on expression quantiles. Hallmark and canonical pathways (MSigDB v7.5.1) were used for gene set enrichment. In total, 1000 permutations were applied, and gene sets with FDR *q* < 0.25 and nominal *P* < 0.05 were considered significant.

### Statistical analysis

Statistical analyses were conducted with GraphPad Prism 9, and results are expressed as mean ± standard deviation (SD). Comparisons between two groups were made using an unpaired *t* test for data with a normal distribution or the Mann–Whitney *U* test for data without normal distribution. In cases involving more than two groups, one-way ANOVA test was not conducted. *P* values were interpreted in the context of these targeted comparisons. Statistical significance was defined as a *P* value of less than 0.05, with significance levels indicated as follows: *P < 0.05, **P < 0.01, ***P < 0.001, and ****P < 0.0001.

## Supplementary information

Supplementary file

## Data Availability

Data are provided within the manuscript or supplementary information files.
