# Supplementary material for: Prkci acts a pro-proliferation factor in colorectal cancer
Source: NPJ Precis Oncol. 2025 Nov 4;9:338. doi: 10.1038/s41698-025-01117-y (PMC12586529; doi:10.1038/s41698-025-01117-y)
Supplement: Supplementary file 1 — Supplementary file [file 41698_2025_1117_MOESM1_ESM.pdf]

1. ARRIVE Essential 10
2. Supplementary figure legend
3. Supplementary figure
4. Original gel

ARRIVE Essential 10

1. Study design

This study aimed to investigate the oncogenic role of Prkci in colorectal cancer (CRC). In vitro experiments were conducted using CRC cell lines with Prkci overexpression or knockout to evaluate proliferation, metabolism, and downstream molecular mechanisms. In vivo, a subcutaneous xenograft model was established using BALB/c nude mice to assess the effect of Prkci depletion on tumor growth and mouse survival. Appropriate control groups were included throughout.

2. Sample size

Five female BALB/c nude mice were used per group (n = 5) for the in vivo xenograft assays. Sample size was determined based on previous experience and experimental reproducibility; no formal sample size calculation was performed.

3. Inclusion and exclusion criteria

Only healthy female BALB/c nude mice aged 6–8 weeks were included. No animals were excluded or died during the experiment. All animals were included in the final data analysis.

4. Randomisation

Mice were randomly assigned to experimental groups using a random number table prior to cell injection to reduce allocation bias.

5. Blinding

All in vivo procedures—including group allocation, cell injection, tumor measurement, survival monitoring, and tissue staining—were performed in a blinded manner. Investigators were unaware of group assignments during the experimental procedures and outcome assessments.

6. Outcome measures

Primary outcome measures included:

In vivo: Tumor volume, tumor weight, survival time;

Tissue level: Immunohistochemical expression of c-Myc and Ki-67 in tumor samples.

7. Statistical methods

Data with normal distribution were analyzed using unpaired two-tailed Student’s t-test;

Data without normal distribution were analyzed using the Mann-Whitney U test; Results are expressed as mean ± standard deviation (SD); All statistical analyses were performed using GraphPad Prism 9; A p-value < 0.05 was considered statistically significant.

8. Experimental animals

Species: BALB/c nude mice (female); Age: 6–8 weeks; Health status: Specific pathogen-free (SPF); Supplier: GemPharmatech Co., Ltd; Number per group: 5 mice; All animal experiments were approved by the Ethics Committee of People's Hospital of Xinjiang Uygur Autonomous Region.

9. Experimental procedures

Each mouse received a subcutaneous injection of 5×10^6 SW48 cells in 100 μL PBS:Matrigel (1:1) mixture into the dorsal flank; Tumor size was measured every 3 days, and tumor volume was calculated using the formula: (length $\times$ width^2) / 2; At the experimental endpoint, mice were euthanized by CO_2 inhalation; All procedures were performed under blinded conditions; No anesthesia was used during cell injection or monitoring; Animal housing, handling, and welfare adhered to institutional and national guidelines.

10. Results

Results are presented in figures with appropriate statistical annotations. All key experiments were performed in biological replicates. In vivo data (e.g., tumor volume, weight, survival) are presented in Figure 7.

Supplementary figure legend

Figure S1. Prkci expression is elevated in kinds of cancer and correlates with tumor progression and patient survival. (A) Analysis of Prkci expression in kinds of cancer tissues using The Cancer Genome Atlas (TCGA) dataset. (B) Survival probability in kinds of cancer types from the TCGA database, patients grouped by Prkci expression levels.

Figure S2. Prkci positively activated c-Myc signaling. (A) Gene Set Enrichment Analysis (GSEA) highlighting activation of c-Myc signaling pathways associated with high Prkci expression. (B) Western blot analysis showing elevated c-Myc, Cdk4, and Glut1 protein levels in vector and Prkci-overexpressing cells. (C) Western blot analysis showing elevated c-Myc, and Cdk4 protein levels in Prkci knockout cells. Each IB assay was performed in triplicate, yielding consistent results.

Figure S3. Prkci did not affect the mRNA expression of c-MYC. (A) RT-PCR analysis showing the mRNA level of c-MYC in different cells. (B) Immunohistochemical (IHC) staining of c-MYC in colorectal cancer (CRC). Statistical analysis was conducted using Student's t-test.

Figure S4. Prkci phosphorylated c-Myc. (A) Western blot analysis of the phosphorylation level of c-Myc in vector and Prkci-overexpressing cells. (B, C) Co-IP of Prkci with different c-Myc segments. (D) Western blot analysis showing the successful construction of Prkci knock-out cell. (E) Western blot analysis showing the successful construction of LoVo stably expressing wild-type c-MYC (WT), S21A, or S21E. Each IB assay was performed in triplicate, yielding consistent results.

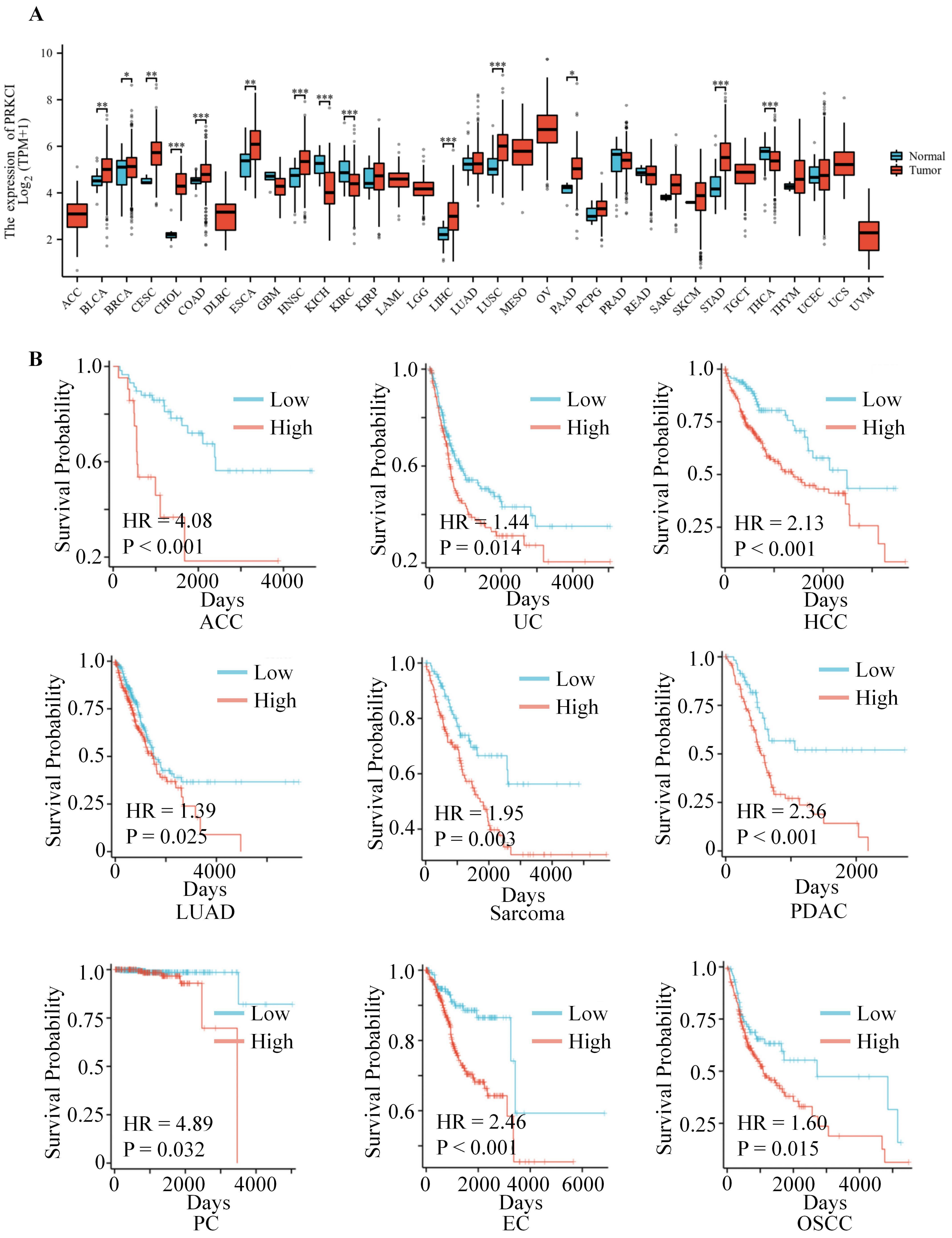

A
The expression of PRKCI
Log_2 (TPM+1)
10
8
6
4
2
Normal
Tumor
ACC
BLCA
BRCA
CESC
CHOL
COAD
DLBC
ESCA
GBM
HNSC
KICH
KIRC
KIRP
LAML
LGG
LIHC
LUAD
LUSC
MESO
OV
PAAD
PCPG
PRAD
READ
SARC
SKCM
STAD
TGCT
THCA
THYM
UCEC
UCS
UVM
B
Survival Probability
Days
Low
High
HR = 4.08
P < 0.001
ACC
HR = 1.44
P = 0.014
UC
HR = 2.13
P < 0.001
HCC
HR = 1.39
P = 0.025
LUAD
HR = 1.95
P = 0.003
Sarcoma
HR = 2.36
P < 0.001
PDAC
HR = 4.89
P = 0.032
PC
HR = 2.46
P < 0.001
EC
HR = 1.60
P = 0.015
OSCC

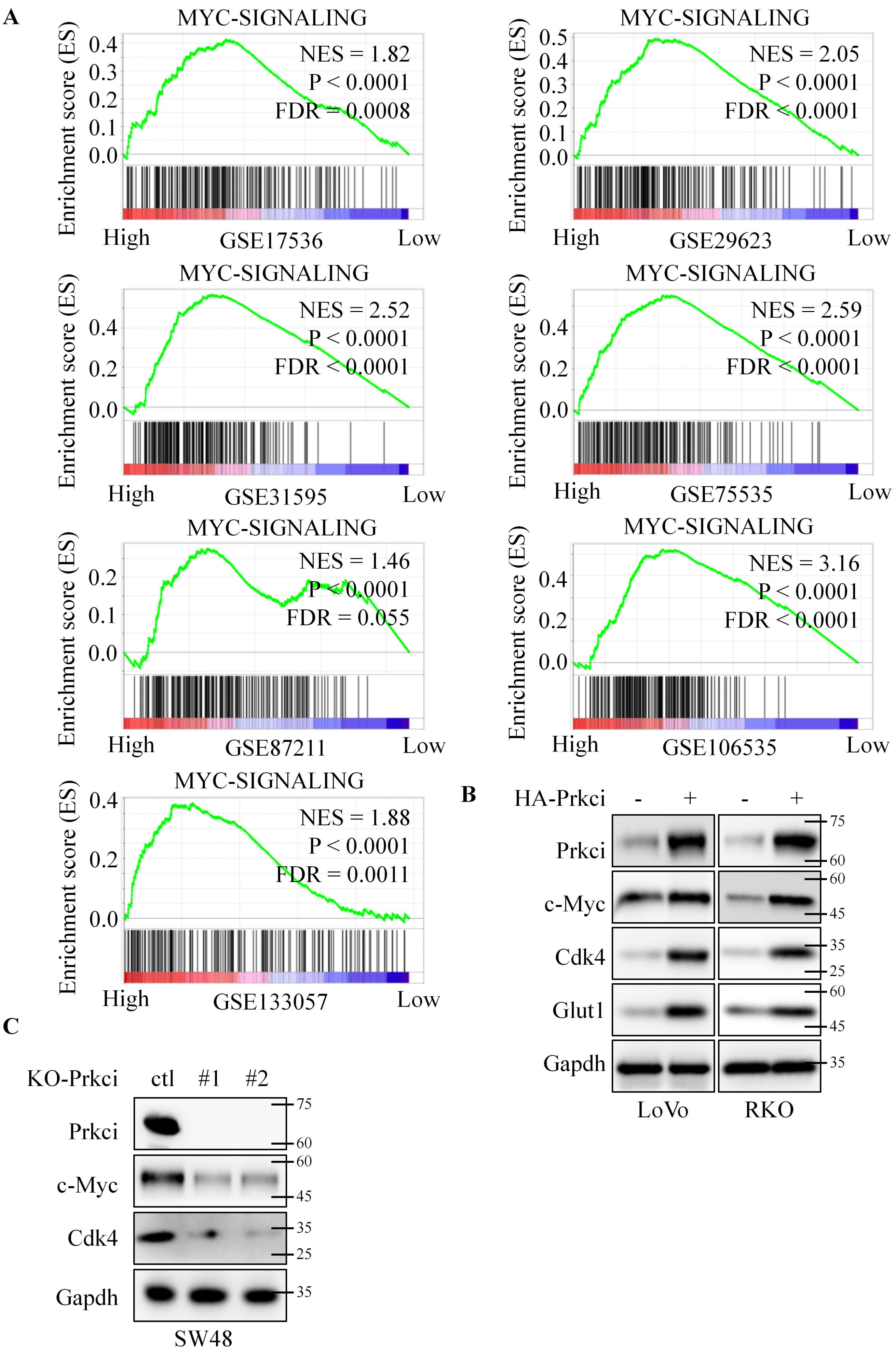
A
MYC-SIGNALING
NES = 1.82
P < 0.0001
FDR = 0.0008
Enrichment score (ES)
High
GSE17536
Low
MYC-SIGNALING
NES = 2.05
P < 0.0001
FDR < 0.0001
High
GSE29623
Low
MYC-SIGNALING
NES = 2.52
P < 0.0001
FDR < 0.0001
High
GSE31595
Low
MYC-SIGNALING
NES = 2.59
P < 0.0001
FDR < 0.0001
High
GSE75535
Low
MYC-SIGNALING
NES = 1.46
P < 0.0001
FDR = 0.055
High
GSE87211
Low
MYC-SIGNALING
NES = 3.16
P < 0.0001
FDR < 0.0001
High
GSE106535
Low
MYC-SIGNALING
NES = 1.88
P < 0.0001
FDR = 0.0011
High
GSE133057
Low
B
HA-Prkci - + - +
Prkci
c-Myc
Cdk4
Glut1
Gapdh
LoVo
RKO
C
KO-Prkci ctl #1 #2
Prkci
c-Myc
Cdk4
Gapdh
SW48

3A

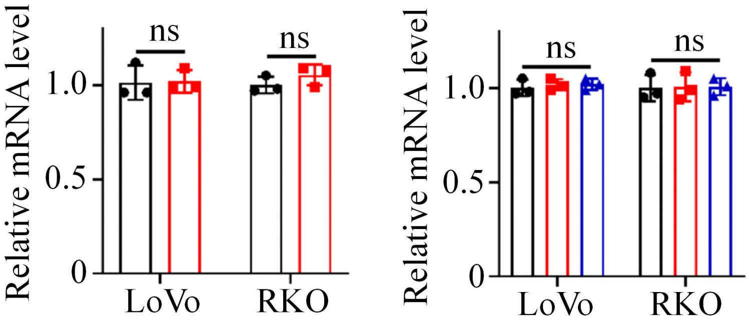
Relative mRNA level
1.0
0.5
0
ns
ns
LoVo
RKO
Relative mRNA level
1.0
0.5
0
ns
ns
LoVo
RKO

3B

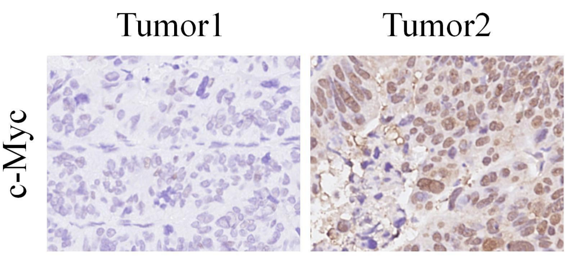
Tumor1
Tumor2
c-Myc

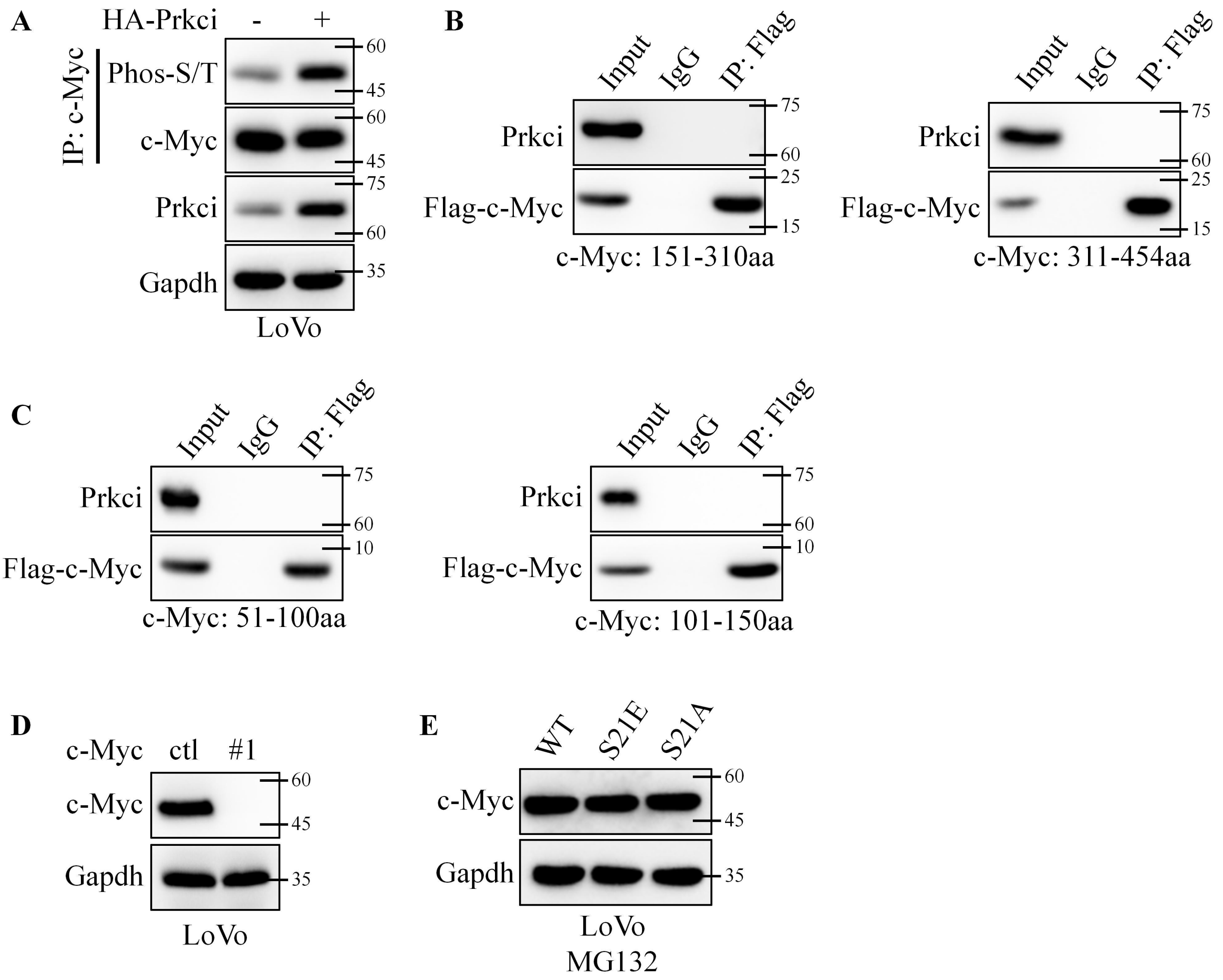

A
HA-Prkci - +
IP: c-Myc
Phos-S/T
c-Myc
Prkci
Gapdh
60
45
60
45
75
60
35
LoVo
B
Input
IgG
IP: Flag
Prkci
Flag-c-Myc
75
60
25
15
c-Myc: 151-310aa
Input
IgG
IP: Flag
Prkci
Flag-c-Myc
75
60
25
15
c-Myc: 311-454aa
C
Input
IgG
IP: Flag
Prkci
Flag-c-Myc
75
60
10
c-Myc: 51-100aa
Input
IgG
IP: Flag
Prkci
Flag-c-Myc
75
60
10
c-Myc: 101-150aa
D
c-Myc ctl #1
c-Myc
Gapdh
60
45
35
LoVo
E
WT
S21E
S21A
c-Myc
Gapdh
60
45
35
LoVo
MG132

Figure 2A

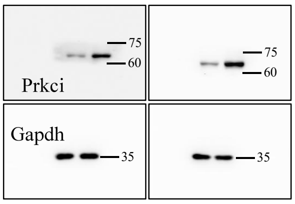

Figure 3A

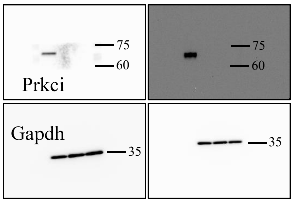

Figure 4C

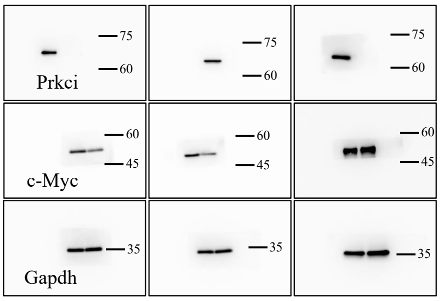

Figure 4B

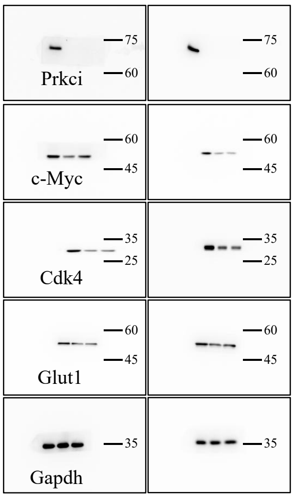

Figure 4F

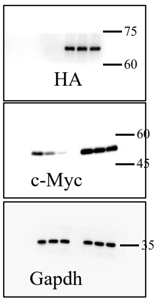

Figure 4D

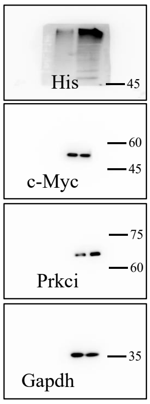

Figure 4E

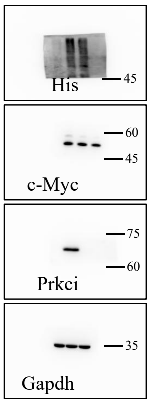

Figure S2B

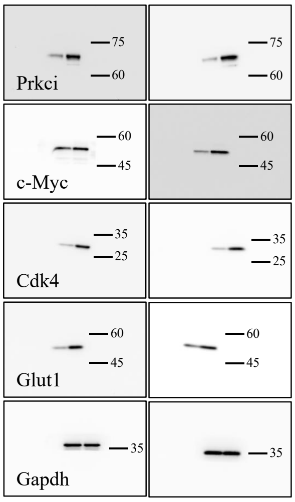

Figure S2C

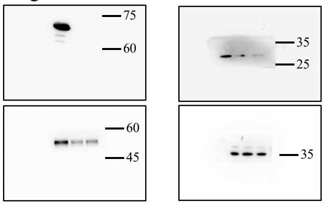

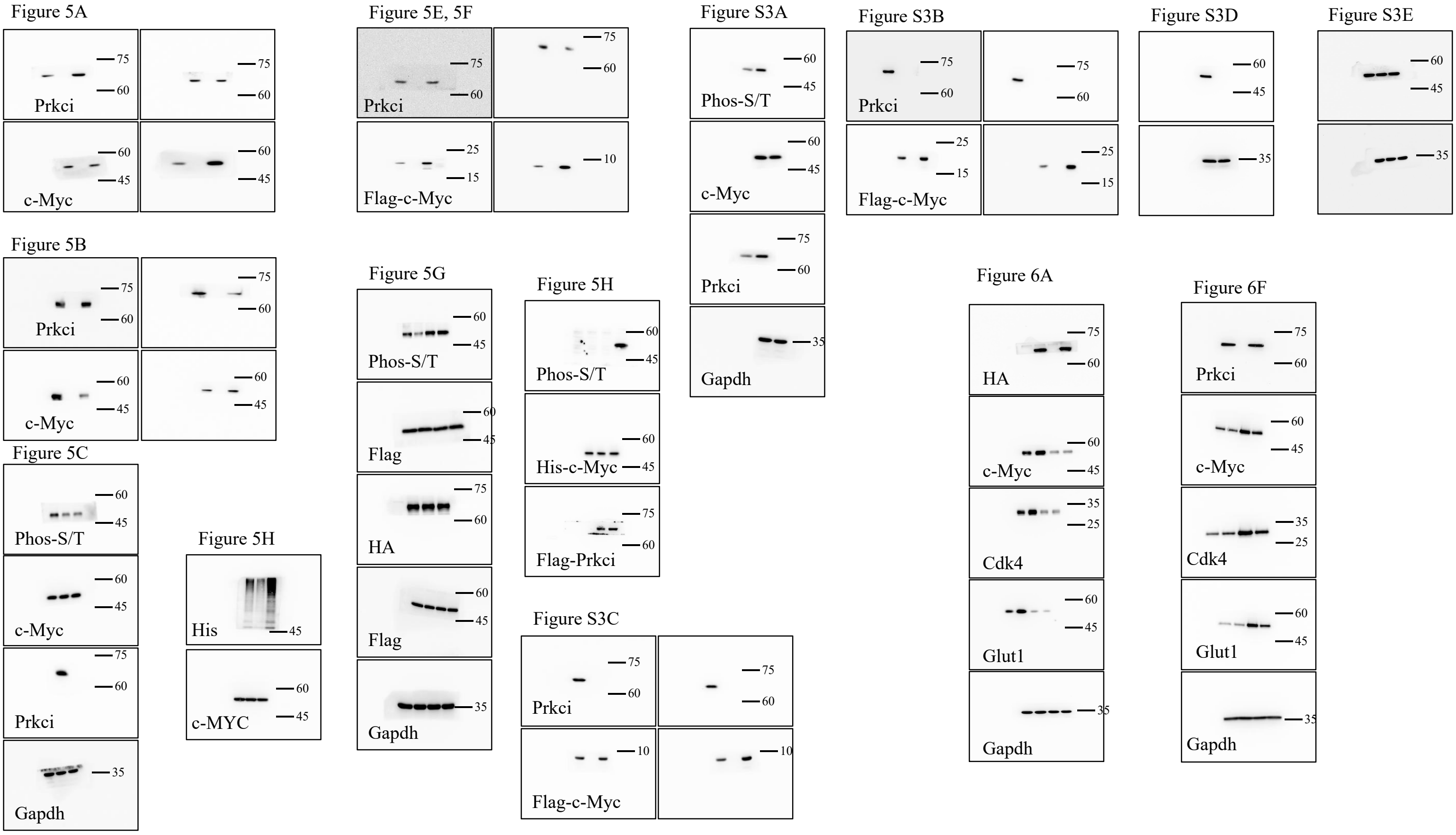
Figure 5A
Prkci
c-Myc
Figure 5E, 5F
Prkci
Flag-c-Myc
Figure S3A
Phos-S/T
c-Myc
Prkci
Gapdh
Figure S3B
Prkci
Flag-c-Myc
Figure S3D
Figure S3E
Figure 5B
Prkci
c-Myc
Figure 5C
Phos-S/T
c-Myc
Prkci
Gapdh
Figure 5G
Phos-S/T
Flag
HA
Flag
Gapdh
Figure 5H
Phos-S/T
His-c-Myc
Flag-Prkci
Figure 5H
His
c-MYC
Figure S3C
Prkci
Flag-c-Myc
Figure 6A
HA
c-Myc
Cdk4
Glut1
Gapdh
Figure 6F
Prkci
c-Myc
Cdk4
Glut1
Gapdh
